# Bacteriophage Resistance, Adhesin’s and Toxin’s Genes Profile of *Staphylococcus aureus* Causing Infections in Children and Adolescents

**DOI:** 10.3390/microorganisms13030484

**Published:** 2025-02-21

**Authors:** Nikolaos Giormezis, Assimina Rechenioti, Konstantinos Doumanas, Christos Sotiropoulos, Fotini Paliogianni, Fevronia Kolonitsiou

**Affiliations:** 1 Department of Microbiology, School of Medicine, University of Patras, 26504 Patras, Greece; 2 Private Microbiological Laboratory, 26225 Patras, Greece

**Keywords:** *Staphylococcus aureus*, bacteriophage K, antimicrobial resistance, PVL, adhesins, toxins, biofilm

## Abstract

*Staphylococcus aureus* is a common pathogen, often recovered from children’s infections. Βiofilm formation, antimicrobial resistance and production of adhesins and toxins contribute to its virulence. As resistance to antimicrobials rises worldwide, alternative therapies like bacteriophages (among them the well-studied Bacteriophage K) can be helpful. The aim of this study was to determine the bacteriophage and antimicrobial susceptibility and the presence of virulence genes among *S. aureus* from infections in children and adolescents. Eighty *S. aureus* isolates were tested for biofilm formation and antimicrobial susceptibility. The presence of two genes of the *ica* operon (*icaA*, *icaD*), adhesin’s (*fnbA*, *fnbB*, *sasG*) and toxin’s genes (*PVL*, *tst*, *eta*, *etb*) was tested by PCRs. Susceptibility to Bacteriophage K was determined using a spot assay. Thirteen isolates were methicillin-resistant (MRSA) and 41 were multi-resistant. Twenty-five *S. aureus* (31.3%) were resistant to Bacteriophage K, mostly from ocular and ear infections. Twelve *S. aureus* (15%) were PVL-positive, seven (8.8%) positive for *tst*, 18 (22.5%) were *eta*-positive and 46 were (57.5%) *etb*-positive. A total of 66 (82.5%) isolates carried *fnbA*, 16 (20%) *fnbB* and 26 (32.5%) *sasG*. PVL, *tst* and *sasG* carriage were more frequent in MRSA. Bacteriophage-susceptible isolates carried more frequently *eta* (32.7%) and *etb* (69.1%) compared to phage-resistant *S. aureus* (0% and 32%, respectively). Although mainly methicillin-sensitive, *S. aureus* from pediatric infections exhibited high antimicrobial resistance and carriage of virulence genes (especially for exfoliative toxins and *fnbA*). MRSA was associated with PVL, *tst* and *sasG* carriage, whereas Bacteriophage susceptibility was associated with *eta* and *etb*. The high level of Bacteriophage K susceptibility highlights its potential use against staphylococcal infections.

## 1. Introduction

*Staphylococcus aureus* is a common pathogen associated with a broad spectrum of pediatric infections ranging from mild, often recurrent, skin, and soft tissue infections (SSTIs) to invasive disease, including bacteremia, necrotizing pneumonia, endocarditis and toxin-mediated disease [1]. Thanks in part to the successful development of vaccines against *Streptococcus pneumoniae* and *Haemophilus influenzae*, *S. aureus* is the leading invasive bacterial pathogen in children in many parts of the world. The emergence of methicillin-resistant *S. aureus* (MRSA) was first described in hospital settings and soon emerged in the community [2]. However, during recent years, several studies have reported the emergence and predominance of methicillin-susceptible isolates (MSSA) [2,3,4]. However, MRSA continues to be a significant cause of hospital and community-acquired infections. According to guidelines from the Expert Panel of the Infectious Diseases Society of America (IDSA) for use by health-care providers who care for adult and pediatric patients with MRSA infections, vancomycin, clindamycin or linezolid can be considered in pediatric cases of skin and soft tissue infections, pneumonia, bacteremia and other types of infection [5].

The capacity of *S. aureus* to cause infection is related to the expression of virulence factors and the production of a wide variety of toxins which include pore-forming molecules, phagocytosis inhibitors and superantigens [6]. Panton–Valentine leukocidin (PVL) is a toxin first described in 1932, composed of two components, LukS-PV and LukF-PV, which lead to neutrophil lysis [7,8]. PVL genes have been linked to community-onset MRSA disease worldwide [9]. Toxic shock syndrome toxin (TSST) is one of the most important *S. aureus* superantigens and acts by stimulating the release of IL-1, IL-2, TNF-α and other cytokines causing toxic shock syndrome [10]. To date, four serotypes of exfoliative toxins have been identified, of which exfoliative toxins A (ETA) and B (ETB) are considered responsible for most human cases of toxin-mediated disease. Exfoliative protein-producing isolates have been associated with a wide range of skin infections, from localized epidermal infections such as impetigo to generalized disease [11,12]. *S. aureus* isolates carrying *eta* are frequently isolated from patients with bullous impetigo, whereas those carrying *etb* are recovered from individuals with Staphylococcal Scalded Skin Syndrome (SSSS). The expression of *eta* and *etb* is positively regulated by common transcription factors (saeR, arlR and agrC); however, the downregulation occurs in different ways. In vivo, *eta* is negatively regulated by transcription factors sarS, while *etb* is downregulated by sarA [13].

Biofilm formation is another important virulence factor based on the attachment, proliferation and maturation of bacteria embedded in a matrix of extracellular polymeric substances. Biofilms play an important role in the pathogenesis of chronic infection, developing and spreading drug resistance through antibiotic impermeability due to the polymer matrix, facilitating gene transfer through conjugation and increasing genetic mutations due to bacterial interactions and the development of isolates with new characteristics. In staphylococci, biofilm formation is associated with the presence of the *ica* operon [14]. The first step to biofilm formation is the bacterial attachment to a surface. Staphylococci are attached to hydrophobic abiotic surfaces by adhesive surface proteins belonging to the Microbial Surface Components Recognizing Adhesive Matrix Molecules (MSCRAMMs), by cell wall anchored proteins and wall teichoic acids. Among the proteins playing an important role in the attachment step are the fibronectin binding proteins A and B (FnbPA, FNbPB) and the surface protein G, known as adhesin SasG [1,15]. The presence of both FnBPs has been associated with severe staphylococcal infections [7].

Bacteriophages are present in nearly every environment and they play an important role in the control of bacterial population. Numerous reports focus on their in vitro application as whole bacteriophages or phage-derived endolysins to treat bacterial infections. Phages with strictly lytic life cycles are preferred for therapeutic implementation, resulting in killing their host since they have been found to diminish the chances of subsequent phage resistance [16]. In this study we have used bacteriophage K, a well-studied anti-staphylococcal phage that attaches specifically to the cell wall teichoic acids, showing a broader host range and having the ability to lyse *S. aureus* and *S. epidermidis*. Bacteriophage K has already been shown to disrupt biofilms produced by *S. aureus* [17].

The aim of this study was the molecular characterization of antimicrobial resistance and virulence factors in *S. aureus* strains (*n* = 80) isolated from hospitalized children and adolescents during the COVID-19 pandemic with a focus on the bacteriophage susceptibility and the carriage of virulence genes, such as genes encoding methicillin-resistance, toxins and adhesins.

## 2. Materials and Methods

### 2.1. Design and Population

This study included all *S. aureus* isolates recovered from children and adolescents younger than 16 years from the University Hospital of Patras, Greece, during the COVID-19 pandemia (2020–2023). The Hospital provides tertiary healthcare services to the Western part of Greece. Infections included skin and soft tissue infections (SSTIs) such as impetigo, cellulitis, and abscesses, as well as other types of infections including pneumonia, bacteremia, ocular and ear infections. Isolates were included if culture type and site were consistent with *S*. *aureus* infection. Staphylococci obtained from anatomic sites not indicative of infection were classified as colonizing isolates and were excluded.

### 2.2. Phenotype Identification and Antibiotic Susceptibility Testing

The isolates were first identified using conventional methods, including colony morphology, Gram staining, standard biochemical tests and finally by Maldi-Tof MS (Bruker, Billerica, MA, USA). Briefly, bacteria were cultured on Mueller–Hinton agar supplemented with 5% sheep blood at 37 °C for 24 h. Pure colonies were applied as a thin film onto a steel plate and allowed to dry at room temperature. Subsequently, 1 μL of matrix (a saturated solution of α-cyano-4-hydroxycinnamic acid in 50% acetonitrile and 2.5% trifluoroacetic acid) was applied onto the colony and allowed to dry before testing. The identification was confirmed by PCR for the species-specific *nuc* gene [18], using the ATCC49775 strain as a positive control. All *S. aureus* isolates were stored in Tryptic Soy Broth with 30% glycerol at −70 °C for further testing. Antimicrobial susceptibilities were determined for penicillin, oxacillin, erythromycin, clindamycin, gentamicin, tobramycin, ciprofloxacin, linezolid, teicoplanin, vancomycin, daptomycin, tetracycline, fusidic acid, rifampicin and trimethoprim/sulfamethoxazole using the disc diffusion method or E-test according to EUCAST guidelines [19]. Isolates were phenotypically classified as MSSA or MRSA based on the cefoxitin disk diffusion test and the latex agglutination test for the detection of PBP2a (bioMerieux, Marcy-l'Étoile, France). Strains showing resistance to at least three classes of antimicrobials were characterized as multi-resistant (MDR). The determination of the multiple antimicrobial resistance (MAR) index (the number of antimicrobials to which an isolate is resistant, divided by the total number of antimicrobials used in the study) was performed as previously described [20].

Biofilm formation was tested using a qualitative (glass tube assay) and a quantitative microtiter plate assay, as previously described [21,22]. The first method is based on the thickness of biofilm layer formation, on the surface of glass tubes after incubation in Tryptic Soy Broth (TSB, Oxoid Ltd., Basingstoke, Hampshire, UK) for 24 h. For the microtiter plate assay, strains were inoculated onto Tryptic Soy agar (TSA, Oxoid Ltd.); after an overnight incubation, three colonies were subcultured in 5 mL TSB and incubated for 18 h; after vortexing, 2 μL of the suspension were inoculated into 200 μL TSB with 1% glucose (Glucose monohydrate, Sigma-Aldrich, St. Louis, MO, USA), in 96 well U-bottomed sterile polystyrene plates that were incubated aerobically at 37 °C for 24 h. After washing each well with 300 μL sterile phosphate-buffered saline (PBS), plates were heat-fixed at 60 °C for 1 h. Staining was performed with 195 μL Crystal violet for 15 min, followed by a washing step with tap water and air drying. The optical density (OD) of each stained well was measured at 570 nm using a microtiter-plate reader (FLUOstar Omega microplate reader, BMG LABTECH GmbH, Ortenberg, Germany, https://www.bmglabtech.com/en/fluostar-omega/, accessed on 6 December 2024), after homogeneous resolubilization of the dye with 150 μL of 95% ethanol. The cut-off value (ODc) in each plate was defined as three standard deviations (SD) above the mean OD of an uninoculated well [22]. Reference strains *S. epidermidis* ATCC35984 (RP62A) and ATCC12228 were used in both assays as positive and negative controls, respectively.

### 2.3. Susceptibility to Bacteriophage K

Susceptibility to Bacteriophage K was determined using a spot assay. The test was performed for each isolate in duplicate, using a phage suspension of 10^5^ pfu/mL. All staphylococci were inoculated onto Brain-Heart Infusion Agar (BHIA) plates and after an overnight incubation, bacterial suspensions equivalent to 0.5 MacFarland turbidity standard were prepared in Brain-Heart Infusion Broth (BHIB). From each isolate’s suspension, 20 μL was added into 180 μL BHIB together with 20 μL of the Bacteriophage K suspension; after gentle agitation, 20 μL of the mix was inoculated onto BHIA plates, left to dry, and incubated overnight at 37 °C. Mean values of plaque forming units and the efficacy of plating (EOP) for each isolate were calculated. Isolates with EOP equal to or lower than 0.001 were considered phage resistant. As a positive control, the propagating strain ATCC19685 was used and as a negative one, a sample without bacterium [23,24].

### 2.4. Molecular Analysis

DNA extraction from studied strains was performed using the QIAamp DNA Mini kit (Qiagen, Düsseldorf, Germany). *S. aureus* isolates that were resistant to cefoxitin were phenotypically classified as MRSA and verified by PCR for *mecA* and *mecC* gene carriage [25,26]. Genes encoding Panton–Valentine leukocidin (*lukS*/*lukF-PV*), toxic shock syndrome toxin-I (*tst*), exfoliative toxins A and B (*eta*, *etb*), fibronectin binding proteins A and B (*fnbA*, *fnbB*), staphylococcal surface protein G (*SasG*) and two genes of the *ica* operon (*icaA*, *icaD*), were detected via polymerase chain reactions with specific primers, as previously described [27,28,29,30]. PCR products were analyzed by electrophoresis into 1% agarose gels (Figure S1).

### 2.5. Statistical Analysis

For categorical variables, chi-square or two-tailed Fisher’s exact tests were conducted to calculate *p* values and odds ratios to identify differences in antimicrobial susceptibility and molecular characteristics of *S. aureus* isolates. The level of significance was set at a *p* value of < 0.05. Statistical analysis was conducted using GraphPad Prism 9.1.0.

## 3. Results

### 3.1. Clinical Characteristics

During the three-year period (2020–2023), 80 non-duplicated *S. aureus* isolates were collected from children and adolescents aged under 16 years with invasive and non-invasive infections. *S.aureus* was isolated more frequently from skin and soft tissue infections (SSTIs; 41/80, 51.3%), followed by ear (14/80, 17.5%) and ocular infections (10/80, 12.5%), cases of pneumonia (8/80, 10%)), bacteremia (3/80, 3.8%), urine (2/80) and catheter-related infections (2/80).

### 3.2. Antibiotic Resistance Profiles

Thirteen (16.3%) isolates were phenotypically identified as MRSA and 41 (51.3%) were classified as multi-resistant. Of the 80 *S. aureus* isolates, 69 (86.3%) were in vitro resistant to penicillin, 45 (56.3%) to fusidic acid, 32 (40%) to tobramycin, 18 (22.5%) to erythromycin, 14 (17.5%) to clindamycin and 9 (11.3%) to gentamicin (Table 1). Low percentages of resistance to tetracycline, ciprofloxacin and trimethoprim/sulfamethoxazole were also observed (8.8%, 3.8% and 3.8%, respectively). All *S. aureus* isolates were susceptible to vancomycin, daptomycin, linezolid or teicoplanin, with MICs ranging between 0.5–2, 0.5–1, 0.19–1.5 and 0.75–1, respectively. All phenotypically cefoxitin-resistant isolates were also oxacillin-resistant, carrying *mecA* (all were *mecC*-negative) and were characterized as MRSA.

### 3.3. Biofilm Formation

Biofilm formation was an uncommon finding in our collection of *S.aureus*; only 4/80 isolates (5%) produced biofilm with the glass tube and the microtiter plate method. In fact, all four isolates were weak biofilm-producers (three from SSTIs and one from an ear infection). One biofilm producer was also MRSA and another one was multi-resistant. All *S. aureus* tested negative for *icaA*, whereas the majority (76/80) carried *icaD*. All biofilm-producing isolates carried *icaD*.

### 3.4. Molecular Characteristics

**Figure 1:**
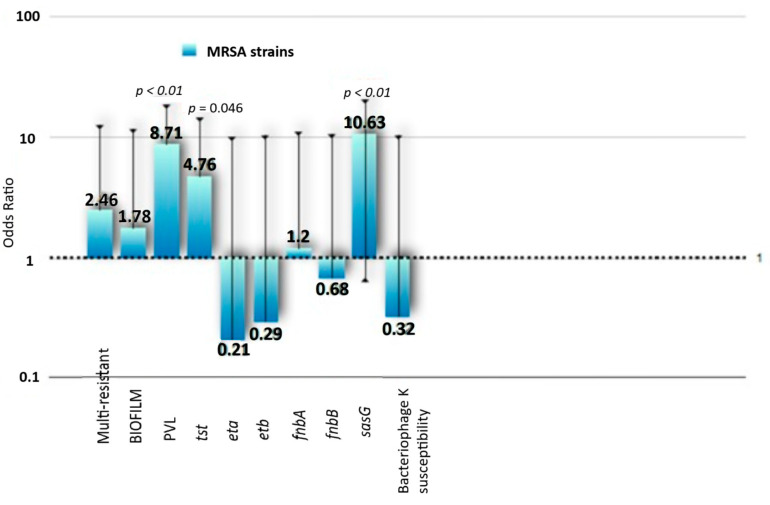
Antimicrobial susceptibility and virulence genes and Bacteriophage K susceptibility among MRSA.

The majority of *S. aureus* (58/80, 72.5%) carried at least one of the tested toxin genes; twelve of the *S. aureus* (15%) were PVL-positive, seven (8.8%) were positive for *tst*, 18 were (22.5%) *eta*-positive and 46 were (57.5%) *etb*-positive. PVL and *tst* carriage were more frequent in MRSA (*p* < 0.001 and *p* = 0.046, respectively, Figure 1). Carriage of both exfoliative toxin’s genes was fairly common in the tested population, since 17/80 (21.3%) isolates in our collection (mostly from SSTIs) were positive for both *eta* and *etb* (Table 2).

The most frequent adhesin was FnbPA. In particular, 66/80 (82.5%) isolates carried *fnbA*, 16 (20%) were *fnbB*-positive and 26 (32.5%) isolates carried *sasG*. The majority of *S. aureus* carried at least one of the tested adhesin’s genes; eleven isolates carried all three genes, whereas 12 isolates carried neither. No association between adhesin’s genes and site of infection was identified. Carriage of *sasG* was associated with resistance to methicillin (*p* < 0.001, Figure 1).

### 3.5. Resistance to Bacteriophage K

**Figure 2:**
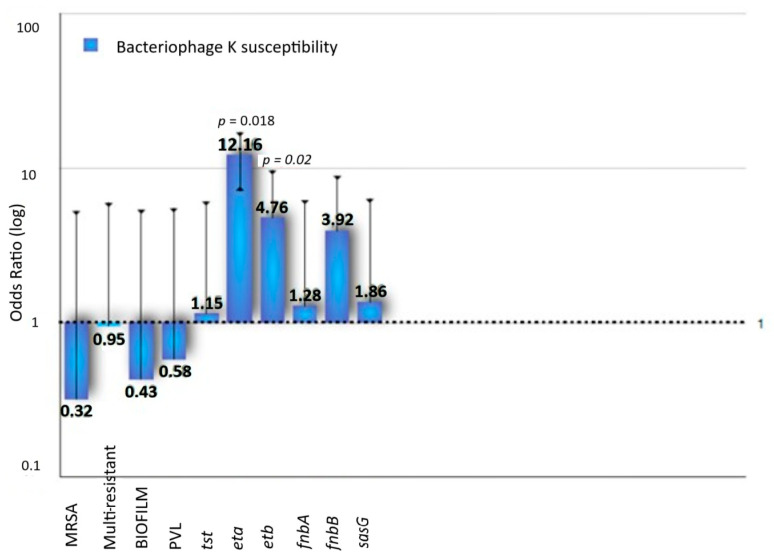
Antimicrobial susceptibility, biofilm formation and carriage of virulence genes among Bacteriophage K-susceptible *S. aureus*.

The spot assay with Bacteriophage K demonstrated that twenty-five *S. aureus* (31.3%) were resistant to Bacteriophage K (EOP < 0.001). Interestingly, most phage-resistant (14/25, 56%) isolates were derived from eye and ear infections (6 and 8 *S. aureus,* respectively). Of the 13 MRSA, the majority (7/13, 53.8%) were phage resistant. Eight isolates (four of which were characterized as MRSA) demonstrated medium susceptibility to Bacteriophage K (EOP between 0.001 and 0.1). Bacteriophage-susceptible isolates carried more frequently *eta* (32.7%) and *etb* (69.1%) in comparison to phage K-resistant *S. aureus* (0% and 32%, respectively, Figure 2).

## 4. Discussion

In the past, Greece has been described as a country with a heavy burden of MRSA infections. Various studies in the last two decades have reported a high incidence of community-acquired MRSA infections in children, largely associated with the spread of PVL positive clones [31]. In a 12-year multicenter study published in 2014, MRSA was shown to be an important cause of both healthcare and community-associated infections in children in Greece [32]. In our patient population, only 16.3% of isolates were MRSA. This agrees with other recent studies, which indicate the current predominance of MSSA in the pediatric population [33]. Most isolates were also multi-resistant, but no resistance to vancomycin, teicoplanin, linezolid and daptomycin was identified. Of the 13 MRSA, nine were also in vitro classified as multi-resistant, as they were resistant to b-lactams, aminoglucosides, fusidic acid and tetracycline. Overall, the studied isolates had MAR indices varying from 0 to 0.47, including 45 *S. aureus* with an MAR index of 0.2 or higher, indicating the extensive use of antibiotics in the clinical setting. The increasing antibiotic resistance worldwide makes empirical antibiotic therapy challenging. A bacterial resistance surveillance program together with improvements in the identification of multi-resistant isolates may further help tailor antimicrobial therapies and avoid unnecessary broad-spectrum antibiotics. Furthermore, combination therapy based on phenotypic and molecular analysis may contribute to optimizing antimicrobial therapy and limiting the emergence of resistance.

Biofilm formation is an important virulence factor for many bacteria, including *S. aureus*. Biofilm development involves the initial attachment, accumulation/maturation, and detachment [34]. In our collection of *S. aureus*, only four isolates tested positive for biofilm formation. This could be attributed to the fact that most *S. aureus* were isolated from SSTIs and other non-invasive infections. The *icaA* gene encodes the enzyme responsible for the polysaccharide biosynthesis and the *icaB* encodes another enzyme for deacetylation of the polysaccharide before binding to the cell surface. Additionally, the *icaD* gene encodes a chaperone to activate other *ica* genes [30]. All *S. aureus* in this study tested negative for *icaA*, in accordance with the low level of detected biofilm production, highlighting the importance of *icaA* for polysaccharide intercellular adhesin synthesis and subsequent biofilm formation.

Toxin production is another important factor in the pathogenesis of staphylococcal infections. The majority of *S. aureus* (72.5%) carried at least one of the tested toxin genes, indicating their virulent potential; more specifically, more than half (57.5%) of the studied isolates carried at least *etb* and 17/80 (21.3%) isolates (mostly from SSTIs) were positive for both exfoliative toxin genes. Exfoliative toxins are an important virulence factor for *S. aureus* and are considered responsible for SSSS, a blistering skin disorder that particularly affects infants and young children, as well as adults with underlying diseases. It has been suggested that damaging the protective epidermis allows the organism to propagate and invade deeper tissues [34]. In our study, most isolates carrying exfoliative genes derived from children with SSTIs, but no case of SSSS was recorded. This could be attributed to the presence of transcription factors that downregulate *eta* and *etb*, as shown by Kato et al. [13]. PVL and TSST-1 were not as common in our collection, since only 12 and 7 isolates carried the respective genes. However, PVL and *tst* carriage were more frequent among MRSA. This finding is in accordance with previous studies linking PVL genes to community-onset MRSA disease worldwide [9]. Evidence that PVL is associated with SSTIs is strong and independent of strain type among both MRSA and MSSA. In fact, it has been reported that individuals with SSTIs associated with PVL-positive isolates are more likely to require surgery and have more recurrent infections than those with PVL-negative infections [35].

To become a pathogen, staphylococci must gain access to the human host usually by adhering to biotic surfaces, such as components of the extracellular matrix or host tissue, or to abiotic surfaces, such as medical devices. Upon adherence, bacteria colonize and proliferate on the respective biotic or abiotic surfaces [36]. Primary attachment to a biotic surface in host tissues and synthetic surfaces coated with plasma proteins, such as fibronectin, fibrinogen and vitronectin is mediated by adhesins like *S. aureus*’ fibronectin-binding protein A and B. *S. aureus* also often colonizes the moist squamous epithelium of the anterior nares. One of the adhesins likely to be responsible is its surface protein G (SasG), which has sequence similarity with the Aap (accumulation-associated protein) of *S. epidermidis* [37]. The most frequent adhesin gene in our study was *fnbA*, followed by *sasG* and *fnbB*. The fact that 68/80 isolates (85%) carried at least one of the adhesin’s genes highlights their important role in the pathogenesis of infection. FnBPA is considered more important for in vitro and in vivo infections; however, cooperation between FnBPA and FnBPB is indispensable for the induction of severe infection resulting in septic death [38].

Carriage of *sasG* was associated with resistance to methicillin (*p* < 0.001). Indeed, *sasG* has previously been associated with endemic and sporadic MRSA from bacteremias and strong biofilm formation, as reported by Carrera-Salinas et al. [39]. The fact that most isolates in this study did not carry *sasG* partially explains the low rate of biofilm detection. Indeed, the importance of *sasG* in biofilm formation has been documented, since SasG-expressing S. *aureus* has been reported to form biofilm independently of the polysaccharide intercellular adhesin (PIA) and that the fibrillar nature of SasG explains its ability to mask the binding of *S. aureus* microbial surface components recognizing adhesive matrix molecules (MSCRAMMs) to their ligands and to promote formation of biofilm [40].

Bacteriophage-based therapies are promising tools in the era of antimicrobial resistance. Antibiotics were first introduced in the 1940s, leading to the belief that bacterial illnesses would be eradicated and the abandonment of traditional practices. However, bacteria, such as *S. aureus*, began to develop antibiotic resistance due to the increased use and abuse of antibiotics. Antibiotic resistance is now one of the most serious threats to global public health. Faced with the critical problem of rising bacterial drug resistance, humanity may confront a “post-antibiotic era” characterized by the absence of effective antibiotics [39]. Meanwhile, the interest towards phage therapy has been rekindled and is gaining renewed attention and development [41]. Although the Food and Drug Administration (FDA) has not yet authorized any phage products for routine clinical use in humans, various studies and phase I/II clinical trials have demonstrated the safety and efficacy of phage therapy [40]. Bacteriophages have a relatively restricted range of action, enabling them to target pathogenic bacteria and leave the rest of the microbiome intact [42]. Bacteriophage K is a member of the *Myoviridae* family and has been the subject of previous preliminary studies which focused on the adsorption and infection processes, the effect of acridines on phage reproduction, morphology, and the nature of phage K DNA replication [43]. In our study, 25 (31.3%) of the *S. aureus* were resistant to Bacteriophage K, indicating that the majority of *S. aureus* from pediatric infections could be eradicated by Phage K as an alternative or complementary treatment. Interestingly, most phage-resistant (14/25, 56%) isolates derived from ocular and ear infections. Another important finding is that Bacteriophage-susceptible isolates carried more frequently *eta* and *etb* in comparison to phage-resistant isolates.

Several in vitro and in vivo studies have reported the potential of phages and their lysins to eradicate MRSA infections [44]. In a study from O’Flaherty et al., phage K was active on 39 of 53 MRSA isolates tested [45]. The combination of Bacteriophage K with antibiotics has also been shown to have antistaphylococcal activity, as has the application of Bacteriophage K to biofilm-infected central venous catheter material, which significantly reduced bacterial colonization and biofilm presence [46]. More recently, an antibacterial implant coating was incorporated into both silicone and titanium alloy implants and loaded with Bacteriophage K; in addition to the desired immunomodulatory properties, this modified coating enabled swift release of Phage K, resulting in the efficient elimination of over 99.99% of *S. aureus* in less than 8 h [47]. From the 13 MRSA in our study, the majority (7/13, 53.8%) were phage-resistant, whereas four MRSA demonstrated a reduced susceptibility to Bacteriophage K with an EOP ranging between 0.001 and 0.1. The combination of Phage K with other bacteriophages, lysins or antibiotics could enhance its efficiency against our clinical MRSA isolates, as shown before [48,49]. For example, combination of Phage K and daptomycin in high concentrations has demonstrated the best results against *S.aureus* and *S. epidermidis* [24].

The strength of this prospective study was the analysis of *S. aureus* isolates, causing infection being restricted to the pediatric population in a time of a pandemia, when research was mostly associated with COVID-19 and related infections. To our knowledge, this is the first study concerning *S. aureus* infection in Western Greece during the pandemic. Limitations of this study include the relatively low number of isolates recovered from a single-center cohort of infected children. However, this also represents the largest tertiary reference center in Western Greece, covering a significant part of the country’s population. Further sequencing-based evaluation would provide insights into the origin of the isolates and the spread of specific clones in children and adolescents.

## 5. Conclusions

Although mainly methicillin-sensitive, *S. aureus* from pediatric infections exhibited a high level of antimicrobial resistance and carriage of virulence genes (especially for exfoliative toxins *eta*, *etb* and *fnbA*). MRSA was associated with PVL, *tst* and *sasG* carriage, whereas susceptibility to Bacteriophage K was associated with *eta* and *etb*. The high level of Bacteriophage K susceptibility indicates its potential use as an alternative therapy against persistent staphylococcal infections.

## Figures and Tables

**Table 1:** Antimicrobial resistance profiles and MAR Index of *S. aureus* isolates to the tested antibiotics (N = 15). PEN: Penicillin, FA: Fusidic acid, ER: Erythromycin, CLI: Clindamycin, TOB: Tobramycin, GM: Gentamicin, TE: Tetracycline, CIP: Ciprofloxacin, FOX: Cefoxitin, SXT: Sulphamethoxazole/Trimethoprim.

Resistance Profile	No of Isolates	MAR Index
PEN, FA, ER, CLI, TOB, GM, TE	1 (1.25%)	0.47
PEN, FA, ER, CLI, TOB, GM	1 (1.25%)	0.4
PEN, FA, ER, CLI, CIP	1 (1.25%)	0.33
PEN, FA, ER, CLI, TOB	1 (1.25%)	0.33
PEN, FA, ER, CLI, FOX	1 (1.25%)	0.33
PEN, FA, FOX, GM, TOB	2 (2.5%)	0.33
PEN, FA, ER, GM, TOB	1 (1.25%)	0.33
PEN, ER, CLI, CIP, TE	1 (1.25%)	0.33
PEN, ER, CLI, TOB	5 (6.25%)	0.27
PEN, FA, TE, FOX	4 (5%)	0.27
PEN, GM, TOB, FOX	2 (2.5%)	0.27
PEN, FA, TOB, GM	1 (1.25%)	0.27
PEN, FOX, FA	3 (3.75%)	0.2
PEN, ER, CLI	2 (2.5%)	0.2
PEN, FA, TOB	14 (17.5%)	0.2
PEN, FA, GM	1 (1.25%)	0.2
PEN, FA, TE	1 (1.25%)	0.2
PEN, TOB, TE	1 (1.25%)	0.2
PEN, FOX, ER	1 (1.25%)	0.2
ER, CLI, TOB	1 (1.25%)	0.2
PEN, FA	12 (15%)	0.13
PEN, TOB	2 (2.5%)	0.13
PEN, SXT	3 (3.75%)	0.13
PEN	8 (10%)	0.07
FA	1 (1.25%)	0.07
ER	2 (2.5%)	0.07
No resistance	7 (8.75%)	0

**Table 2:** Combinations of virulence genes among the studied isolates.

Combination of Virulence Genes	No of Isolates (%)
Toxins	
*PVL*	8 (10%)
*tst*	3 (3.8%)
*eta*	1 (1.3%)
*etb*	22 (27.5%)
*eta*, *etb*	17 (21.3%)
*PVL*, *etb*	3 (3.8%)
*tst*, *etb*	3 (3.8%)
*PVL*, *tst*, *etb*	1 (1.3%)
None	22 (27.5%)
Adhesins	
*fnbA*	38 (47.5%)
*sasG*	2 (2.5%)
*fnbA*, *fnbB*	4 (5%)
*fnbA*, *sasG*	12 (15%)
*fnbA*, *fnbB*, *sasG*	12 (15%)
None	12 (15%)

## Data Availability

All data used and analyzed in the current study will be made available on reasonable request.

## Supplementary Materials

The following supporting information can be downloaded at: https://www.mdpi.com/article/10.3390/microorganisms13030484/s1, Figure S1: Image of a gel showing the PCR amplicons; Lanes 1, 23: DNA ladder 100 bp, Lanes 2, 5, 8, 11, 14, 17, 20, 24, 27: Samples positive for *eta*, *etb*, *tst*, *PVL*, *fnbA*, *fnbB*, *sasG*, *icaA*, *icaD*, respectively. Lanes 3, 6, 8, 12, 15, 18, 21, 25, 28: Positive controls for *eta*, *etb*, *tst*, *PVL*, *fnbA*, *fnbB*, *sasG*, *icaA*, *icaD*, respectively. Lanes 4, 7, 9, 13, 16, 19, 22, 26, 29: Negative controls.
