# Supplementary figures and images for: Bacteriophage Resistance, Adhesin’s and Toxin’s Genes Profile of Staphylococcus aureus Causing Infections in Children and Adolescents
Source: Microorganisms. 2025 Feb 21;13(3):484. doi: 10.3390/microorganisms13030484 (PMC11946024; doi:10.3390/microorganisms13030484)

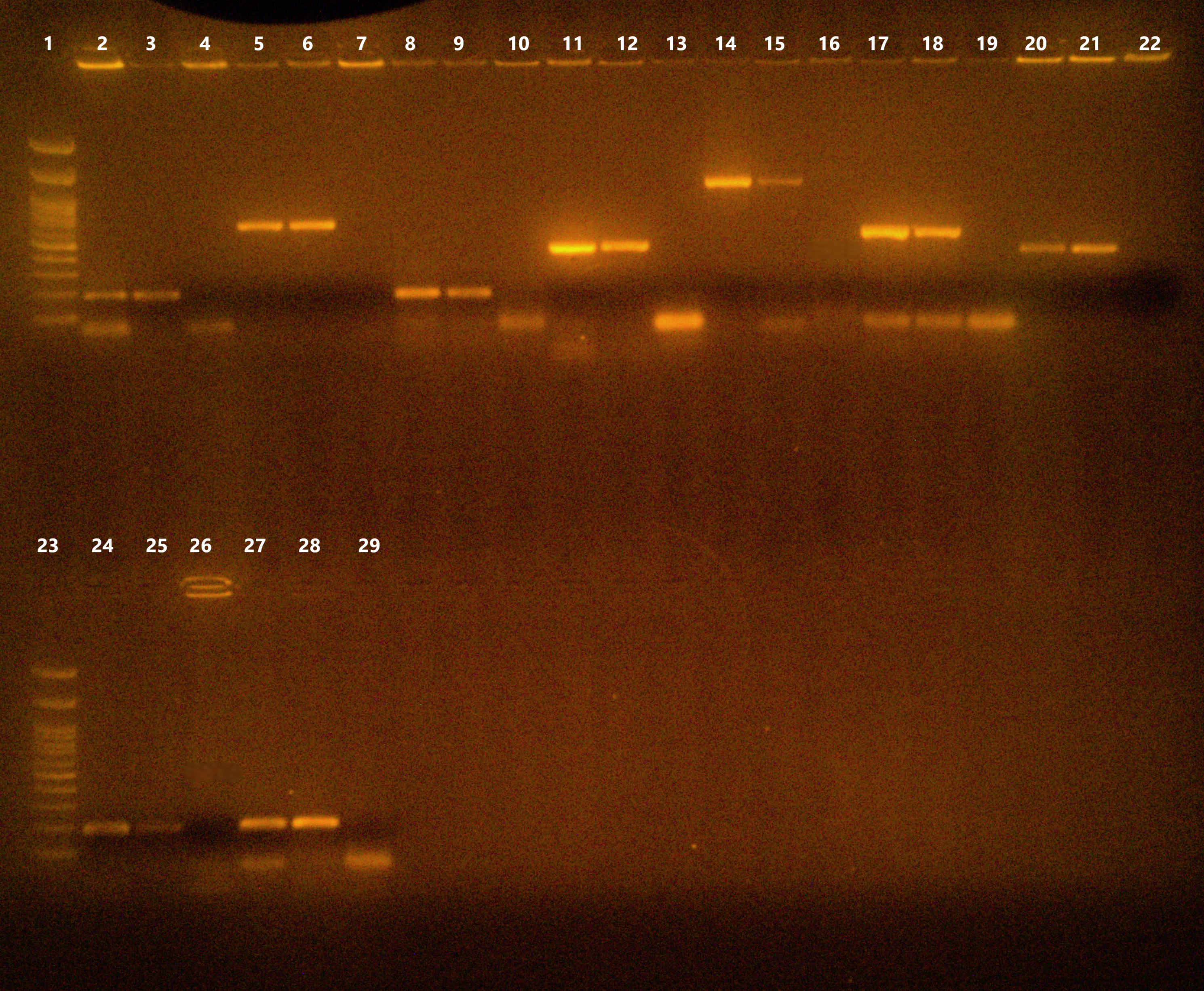

Supplement: Supplementary file 1 [file microorganisms-13-00484-s001.zip › microorganisms-3479937-supplementary.png]
